# The Impact of Electronic Data to Capture Qualitative Comments in a Competency-Based Assessment System

**DOI:** 10.7759/cureus.23480

**Published:** 2022-03-25

**Authors:** Teresa M Chan, Stefanie S Sebok-Syer, Yusuf Yilmaz, Sandra Monteiro

**Affiliations:** 1 Emergency Medicine, McMaster University, Hamilton, CAN; 2 Continuing Professional Development, McMaster University, Hamilton, CAN; 3 Emergency Medicine, Stanford University Medical Center, Palo Alto, USA; 4 Medical Education, Faculty of Medicine, Ege University, Izmir, TUR; 5 Health Research Methods, Evidence and Impact, McMaster University, Hamilton, CAN

**Keywords:** paper-based comment, electronic comment, digitizing, realist evaluation framework, workplace-based assessment

## Abstract

Introduction

Digitalizing workplace-based assessments (WBA) holds the potential for facilitating feedback and performance review, wherein we can easily record, store, and analyze data in real time. When digitizing assessment systems, however, it is unclear what is gained and lost in the message as a result of the change in medium. This study evaluates the quality of comments generated in paper vs. electronic media and the influence of an assessor’s seniority.

Methods

Using a realist evaluation framework, a retrospective database review was conducted with paper-based and electronic medium comments. A sample of assessments was examined to determine any influence of the medium on the word count and the Quality of Assessment for Learning (QuAL) score. A correlation analysis evaluated the relationship between word count and QuAL score. Separate univariate analyses of variance (ANOVAs) were used to examine the influence of the assessor's seniority and medium on word count, QuAL score, and WBA scores.

Results

The analysis included a total of 1,825 records. The average word count for the electronic comments (M=16) was significantly higher than the paper version (M=12; p=0.01). Longer comments positively correlated with QuAL score (r=0.2). Paper-based comments received lower QuAL scores (0.41) compared to electronic (0.51; p<0.01). Years in practice was negatively correlated with QuAL score (r=-0.08; p<0.001) as was word count (r=-0.2; p<0.001).

Conclusion

Digitization of WBAs increased the length of comments and did not appear to jeopardize the quality of WBAs; these results indicate higher-quality assessment data. True digital transformation may be possible by harnessing trainee data repositories and repurposing them to analyze for faculty-relevant metrics.

## Introduction

Electronic data capture systems, which are the new normal at most of the educational institutions that collect assessment data about trainees [1,2], require careful transition planning and change management planning [3,4]. While the previous studies provide some good examples of transitions from a technical standpoint, there may be important individual differences in how faculty adapt to these changes, which may, in turn, lead to different interpretations of educational assessment outcomes [1-4].

Electronic assessment records have the potential to drastically improve assessment data aggregation. If properly implemented, they hold the potential for facilitating feedback and performance review, wherein we can easily record, store, and analyze data in real time [1,5]. Sentiment analysis or natural language machine learning algorithms hold great promise in helping to enhance real-time qualitative analysis as well [6,7], but these technologies are contingent on raters submitting high-quality observations and assessments. Specifically, faculty may differ in how they interact with electronic assessment platforms when recording feedback to trainees [8]. Critically, the technology used to gather and house assessment data may influence the rater-trainee experience. It is well described that electronic medical records (EMRs), for instance, have greatly changed the physician-patient relationship [9]. Similarly, workplace-based assessment (WBA) systems will likely affect the bedside learning environment for both teachers and trainees. For example, paper-based assessments may enhance the timeliness of feedback at the bedside (e.g., Entrustable Professional Activities, Daily Encounter Cards) but are notoriously cumbersome to aggregate [10]. Certainly, communication scientists have established that the change in a medium can greatly affect the message contained within, both in the way that people communicate these messages and the way that they are received [11]. Thus, the bigger challenge is ensuring robust data collection that is not subject to external influences, such as an awkward data entry system or technological barriers. While these considerations are important as part of routine quality assurance processes, strong anecdotal evidence highlighted a need to investigate the contextual influence of the medium on the quality of the feedback messages.

The McMaster Modular Assessment Program (McMAP) was created as a workplace-based assessment program by the emergency medicine training program as a pilot competency-based medical education program [12]. With three levels (junior, intermediate, and senior) of progressively difficult task-based assessments, this program scaffolded tailored learning experiences and competency-focused instruction for trainees in a planned progression within a program of assessment [12]. After a year of piloting a WBA program of assessment with paper booklets in 2012-2013 [12], our program transitioned away from paper to an electronic version of data collection for logging our WBAs in July 2013 with customized branching online data collection forms via our institutional Medportal system (powered by Google Forms™ (Google LLC, Mountain View, California, United States)). Each trainee had their own suite of personalized Google Forms to collect workplace-based assessments, which were then managed manually by our lead designer (TC).

After the transition to the new electronic system, in a subsequent quality improvement focus group, our local residents postulated that the implementation of an electronic system changed the nature of the feedback they received. Specific concerns were raised that more senior faculty provided fewer detailed comments within an electronic platform compared to the previous paper-based platform [8]. At first glance, this concern contradicts recent work. Govaerts and colleagues have noted the effects of a rater’s expertise on assessments; namely that with more complex behaviors, experienced raters tended to take longer to consider the information, searching for additional cues and observing trainees for longer [13]. Experienced raters tended to provide more interpretative, inferential judgments, whereas novice raters tended to provide more literal descriptions. Also, expert raters were thought to have superior abilities to analyze and evaluate contextual or situation-related cues [13]. However, it is unclear whether the more thoughtful analytic approach of expert raters is reflected in word count and comment quality.

The main purpose of the current study was to evaluate if the quality of comments generated in paper vs. electronic media was influenced by an assessor’s seniority. Specifically, we sought to examine the effect of seniority on the quantity (as measured by word count) and quality of written feedback, as assessed using the Quality of Assessment for Learning (QuAL) rubric [14]. We evaluated whether this influence differed for paper and electronic assessments.

## Materials and methods

We adopted a realist evaluation perspective to examine our local workplace-based assessment program [15]. The goal of a realist evaluation framework is to take into consideration how a program’s implementation is affected by contextual factors and how that context works in conjunction with a given mechanism to result in outcomes. Locally, at McMaster University’s specialist emergency medicine program, we have developed a daily WBA program known as the McMaster Modular Assessment Program (McMAP) [12]. Details regarding our successful blueprinting and implementation are discussed elsewhere [12,16-18]. The current study contributes to continued quality assurance and explores the contextual influence of converting McMAP to computer-based data collection methods. We are pleased to report that the previous version of this work was presented as a virtual poster at the Association for Medical Education in Europe (AMEE) 2020 virtual conference.

Procedure

We retrospectively examined the assessments from 85 raters on 30 residents from October 2012 to June 2015. The data from these assessments were preprocessed to anonymize the comments, names, and any user identifier and securely stored in our lead author's encrypted university computer. Comments were then evaluated to objectively determine a quality score and word count.

Data selection

The comments from October 2012 to June 2013 were collected on a paper-based series of WBA workbooks, which were filled out contemporaneously in the clinical setting. The comments from July 2013 to June 2015 were collected using an online e-portfolio system created and housed locally at McMaster University. All data were entered into a Microsoft Excel workbook (Microsoft Corp., Seattle, WA). Each comment examined was associated with a word count, which was determined using standard data processing functions within Excel. To calculate the number of words using Excel, we first removed double spaces and counted the spaces in the comment and added one. Records with zero to three words were excluded from this study. Appendix Table 3 outlines the comments that only had zero to three words so that our readers can appraise their worth as needed.

Coding

There were two independent variables: method of data capture and years in practice. Method of data capture was medium, coded as paper or electronic entry. Years in practice was coded as a continuous variable. Dependent variables were word count, coded as a count for each comment, and comment quality, coded using the QuAL score [14] and McMAP global scale [12], which is a global rating scale of trainee performance on a particular shift rated out of seven on behavioral anchors. Average word counts were rounded and reported as whole numbers [12]. As a result of excluding records with lower word counts, there were no missing data for word count or for QuAL score. However, there were seven missing McMAP global scores, all of which occurred within the paper-based iteration of our system (since the mandatory fields allowed us to prevent such missing data in the electronic version).

Quality Assessment

We used a novel evaluation tool [14] to determine the quality of the assessment comments. The derivation of that tool is described elsewhere [14]. Essentially, three authors (TC, SS, and SM) scored different subsets of the comments. In a previous study, this scoring process was calibrated using 10% of the dataset, achieving an intraclass correlation coefficient (ICC) of 0.95 [14].

Quantitative analysis

All data were processed and coded using Microsoft Excel 2011 for Mac Descriptive Statistics; Pearson correlation, Chi-squared, and univariate analysis of variance (ANOVA) were calculated using IBM SPSS 26 (IBM Corp. Released 2019, IBM SPSS Statistics for Windows, Version 26.0. Armonk, NY: IBM Corp). To evaluate the impact of medium and years in practice on the three dependent variables, word count, QuAL score, and McMAP rating scores were analyzed separately. Pearson correlation analyses were used to describe the relationship between our dependent variables (word count, McMAP score, and QuAL score) and years in practice. Separate univariate ANOVAs were used to describe the impact of medium (paper or electronic) on our dependent variables (word count, McMAP score, and QuAL score). Chi-squared analysis was used to evaluate the independence of frequencies for records with three words or less between paper and electronic entries.

Ethics

Our project was reviewed by our local institutional review board’s chairperson (Hamilton Integrated Research Ethics Board) and granted an exemption.

## Results

From October 2012 to July 2014, 2,556 assessments were generated using written comments, rating scale scores, or both. A total of 1,018 of these assessments were generated within paper workbooks and subsequently transcribed by an administrative staff member or junior faculty member. Of those 1,018 paper-based records, 33% (n=338) were excluded for having three words or less and 22% (n=225) records had no words. From July 2013 to July 2015, daily evaluations of both tasks and overall performance were collected via a novel electronic platform, yielding 1,538 assessments, which included both written comments and numerical ratings. Of those 1,538 records, 24% (n=373) were excluded for having three words or less and 16% (n=241) had zero words. Table 1 shows the distribution of records by year and medium. A Chi-squared analysis of the test of independence of the distribution of comments with less than three words was not significant (χ^2^=2.2, p=0.5). Assessments were from a total of 86 faculty members of whom 64% were male. Around 1% (20) of entries that met the inclusion criteria did not provide information about years in independent practice or rater identity; these records were excluded from the analysis. Table 2 shows the overall demographics of the assessors.

**Table 1:** Yearly distribution of comments with five words or greater n=1,825.

Year	Electronic	Paper
2015	302	0
2014	722	0
2013	136	573
2012	0	92

**Table 2:** Participant demographics and dataset details

	n	%
Gender of trainees		
Female	14	46.7
Male	16	53.3
Gender of raters		
Female	31	36.0
Male	55	64.0
Years in practice		
>20	10	13.9
11 to 20	18	20.9
≤10 yrs (including senior trainees)	58	67.4
Assessment type		
Paper	665	36.4
Electronic	1,160	63.6

Word count

After the exclusion of 731 records, the average word count was 15 (SD=14) across 1,825 comments. There was a significant main effect of medium on word count (F (1,1823)=52.87, p=0.01, partial η^2^=0.83). The electronic records had a higher word count (16) on average compared to paper-based records (12).

Word count and years in practice

Years in practice was negatively correlated to word count (r=-0.2; p<0.001). The correlation was the same when examining paper records and electronic records (for paper: r=-0.25, p<0.001; for electronic r=-0.19, p<0.001).

Quality assessment of comments

The average QuAL score, 0.47/5 (SD=0.86), was lower compared to the sample studied previously (mean=0.9/5, SD=0.9) [14]. The lower range of scores is consistent with prior work, indicating the continued need for faculty development when providing written feedback [14]. Evaluating the transition from paper to electronic data capture, the univariate ANOVA showed a main effect of medium (F (1, 1823)=6.7, p<0.01, partial η^2^=0.004). Critically, for our study goal, there was a small effect size, and the quality of comments was not reduced because of the transition, as the average QuAL score for electronically captured assessments was 0.51 compared to 0.41 for paper-based assessments.

Unsurprisingly, longer comments (regardless of the medium) were positively correlated with scores on the first QuAL subscale (evidence of observed behavior; r=0.46, p<0.01), positively correlated with scores on the second subscale (suggestion for improvement; r=0.40, p<0.01), and positively correlated with scores on the third subscale (evidence linked to suggestion; r=0.41, p<0.001).

Quality assessment of comments and years in practice

Years in practice was negatively correlated with QuAL score (r=-0.08, p<0.001).

McMAP global rating score

The mean McMAP score was 5.9/7 (SD=0.82), with a median score of 6. There was no main effect of medium on McMAP scores.

McMAP global rating score and years in practice

**Figure 1:**
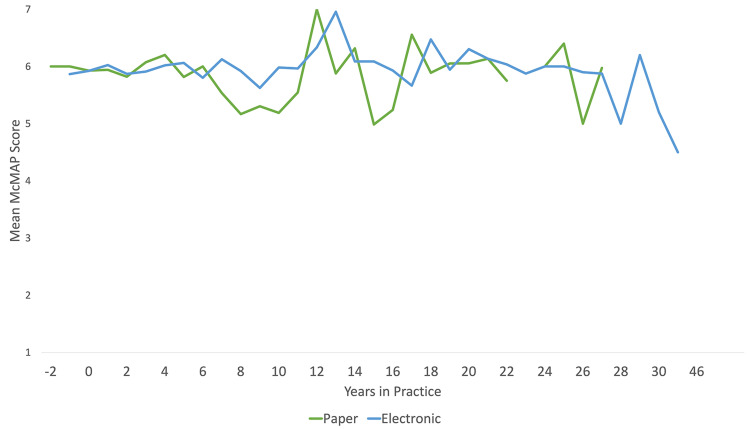
Mean McMAP scores of trainees generated in both contexts (paper vs. electronic) Year 0 on the x-axis denotes the years since the end of their postgraduate training. Numbers less than zero (e.g., negative) on this axis denotes the number of years prelicensure for an individual since senior residents often acted as assessors for junior residents (e.g., senior emergency medicine resident would observe, provide feedback, and rate a first- or second-year trainee).

There was no significant correlation between years in practice and McMAP global rating score. Although Figure 1 depicts some variability in scores across different years in practice, we did not detect a significant or meaningful relationship between years in practice and McMAP scores.

## Discussion

Our study examined the relationship between the method of collecting assessment data and the quality and quantity of words within the comments for one WBA system. You will recall that our intention was to determine if the quality of comments generated in paper vs. electronic media was influenced by an assessor’s seniority. Contrary to the postulations of our residents in our previous qualitative program evaluation study [8], our faculty members were not deterred by the transition to electronic media and, on average, wrote more words for qualitative comments. Faculty members did skip comment boxes more often in the electronic version (23% vs. 9.2%), despite that there were mandatory fields within the digital version.

Going beyond the initial mandate of our study, we also elucidated an interesting finding with regards to the volume of feedback generated by different cohorts of our attending physicians. Mid-career faculty members tended to write the least. In our locale, we hypothesize that the phenomenon we observed may be due to the effects described by Govaerts and colleagues [13], but the phenomenon may also intersect with faculty engagement. In our local quality assurance focus groups, residents revealed that many faculty members were rather disengaged with the new WBA system (McMAP) [8]. As such, we postulate that a number of different forces may be at play.

With the advent of competency-based medical education (CBME), there has been a marked use of digital systems to capture WBA [19-22]. With the increasing use of these databases, many groups have resorted to trainee behaviors around data capture [23,24]. However, more attention must be paid to how faculty respond and engage with these systems and then how faculty respond to their needs via faculty development [25]. Whereas in traditional testing, validity lies in the hands of the students and their engagement in the response process, in the age of CBME and WBAs, the response process of faculty members who enter the data is of paramount importance. Our present study sheds light on an important aspect of the response process for generating high-quality data about trainee performance. Others have examined time burden on faculty [26] with WBA, engagement of faculty [27] in the assessment process, biases they exhibit [28], and even perceptions of their role within these systems [16,17].

In our study, by examining the WBA participation of various faculty cohorts, we show how we might bring more nuanced analyses around different needs of various subgroups of faculty. By doing this type of analysis, we feel that we could begin to refine approaches for faculty development. Rather than seeing faculty as one singular group, more nuanced and targeted approaches to faculty development can be generated by transforming trainee databases to reveal new insights about faculty performance [29].

Next Steps

Trainee databases and repositories may represent a wealth of untapped data that can provide faculty with tangible, actionable insights about their own performance as faculty raters within a system of assessment. A true digital transformation of faculty development may be possible if we harness the newly developed trainee assessment databases to generate useful metrics on faculty performance in terms of their contributions to assessment, feedback, and rating of trainees in the age of CBME [30]. Repurposing trainee data for faculty development insights holds great potential for providing true insights into actual faculty performance related to assessment and their tangible contributions to academic medicine. Future studies in this area may include studies that examine sentiment analysis or applications of natural language processing (such as sequencing of feedback statements, syntactic complexity, local or text coherence, lexical sophistication) to the real-time data capture of trainee feedback comments.

Limitations

This study has a number of limitations. This is a retrospective program evaluation study. Novelty effects of technology may have distorted the use of electronic vs. paper. However, the increased use of the electronic medium can reduce the technological barrier for the raters and create better buy-in to generate feedback to residents. The paper version of data was only from year one, so the score changes from year to year may reflect increasing score drift due to increased usage by faculty members. Therefore, we cannot tease apart the initial pilot year’s novelty effect on our present study. Finally, the data are coming from a single institution with a focus on emergency medicine. The generalizability of our results is limited to our research population.

## Conclusions

We detail our journey through the effective digitalization of a WBA system, which resulted in more words written per comment about trainee performance, and the presence of higher-quality comments. True digital transformation may be possible by harnessing trainee data repositories and repurposing them to analyze for faculty-relevant metrics. Digitalization of workplace-based assessments resulted in an increase in the length of comments available to educators. Longer comments achieved on digital systems did not appear to negatively impact the quality of the assessments. The medium, electronic vs. paper, has a high influence on the evaluative message being sent or received.

## Appendices

**Table 3:** Study dataset Above are the comments for each of our excluded comments (e.g., less than four words). Please note that some of the fields were blank (in paper format) and were bypassed in the electronic format (by placing a space " ") to bypass the mandatory field requirements. The "??" indicates a word that our transcribers could not discern from the handwritten comments. The following quotes are direct verbatim statements written by our raters. The following are definitions that we have inferred but not confirmed: McMAP: McMaster Modular Assessment Program, QuAL: Quality of Assessment of Learning, n/a: not applicable, H/P/P/A: History/Physical/Plan/Assessment, RI: first-year resident, PGY: postgraduate year, ER: emergency room, prev.: previous, mgmt: management, dept: department.

ID	Comment	Word Count	McMAP Score	QuAL Score	Paper = 1; Electronic =0
1	n/a	0	6	0	1
2	n/a	0	6	0	0
3	n/a	0	6	0	1
4		0		0	1
5	n/a	0	6	0	0
6	n/a	0	7	0	0
7	n/a	0	6	0	0
8	n/a	0	6	0	1
9	n/a	0	6	0	1
10	n/a	0	6	0	1
11	n/a	0	6	0	1
12	n/a	0	6	0	1
13	n/a	0	6	0	1
14	n/a	0	5	0	1
15	n/a	0	6	0	1
16	n/a	0	6	0	1
17	n/a	0	6	0	1
18	n/a	0	6	0	1
19	n/a	0	6	0	1
20	n/a	0	6	0	0
21	n/a	0	6	0	1
22	n/a	0	6	0	1
23	n/a	0	6	0	1
24	n/a	0	6	0	1
25	n/a	0	6	0	1
26	n/a	0	6	0	1
27	n/a	0	6	0	1
28	n/a	0	6	0	1
29	n/a	0	6	0	1
30	n/a	0	6	0	1
31	n/a	0	6	0	0
32	n/a	0	6	0	0
33	n/a	0	6	0	1
34	n/a	0	6	0	1
35	n/a	0	6	0	1
36	n/a	0	6	0	1
37	n/a	0	6	0	1
38	n/a	0	5	0	1
39	n/a	0	6	0	1
40	n/a	0	6	0	1
41	n/a	0	6	0	1
42	n/a	0	5	0	1
43	n/a	0	5	0	1
44	n/a	0	6	0	0
45	n/a	0	7	0	0
46	n/a	0	7	0	0
47	n/a	0	7	0	0
48	n/a	0	5	0	0
49	n/a	0	6	0	0
50	n/a	0	6	0	0
51	n/a	0	6	0	0
52	n/a	0	6	0	0
53	n/a	0	6	0	0
54	n/a	0	6	0	0
55	n/a	0	6	0	0
56	n/a	0	7	0	0
57	n/a	0	5	0	0
58	n/a	0	6	0	0
59	n/a	0	5	0	0
60	n/a	0	6	0	0
61	n/a	0	6	0	0
62	n/a	0	6	0	0
63	n/a	0	6	0	0
64	n/a	0	7	0	0
65	n/a	0	7	0	0
66	n/a	0	7	0	0
67	n/a	0	5	0	0
68	n/a	0	6	0	0
69	n/a	0	6	0	0
70	n/a	0	6	0	0
71	n/a	0	6	0	0
72	n/a	0	6	0	0
73	n/a	0	6	0	0
74	n/a	0	6	0	0
75	n/a	0	6	0	0
76	n/a	0	6	0	0
77	n/a	0	6	0	1
78	n/a	0	7	0	0
79	n/a	0	6	0	1
80	n/a	0	6	0	0
81	n/a	0	6	0	1
82	n/a	0	6	0	0
83	n/a	0	6	0	0
84	n/a	0	6	0	0
85	n/a	0	6	0	0
86	n/a	0	5	0	0
87	n/a	0	6	0	0
88	n/a	0	6	0	0
89	n/a	0	6	0	0
90	n/a	0	6	0	0
91	n/a	0	6	0	0
92	n/a	0	6	0	0
93	n/a	0	6	0	0
94	n/a	0	6	0	0
95	n/a	0	6	0	0
96	n/a	0	5	0	0
97	n/a	0	6	0	0
98	n/a	0	5	0	0
99	n/a	0	5	0	0
100	n/a	0	6	0	0
101	n/a	0	6	0	0
102	n/a	0	6	0	0
103	n/a	0	6	0	0
104	n/a	0	5	0	0
105	n/a	0	5	0	0
106	n/a	0	6	0	0
107	n/a	0	5	0	0
108	n/a	0	6	0	0
109	n/a	0	6	0	0
110	n/a	0	6	0	0
111	n/a	0	4	0	1
112	n/a	0	7	0	1
113	n/a	0	5.5	0	1
114	n/a	0	5	0	1
115	n/a	0	6	0	0
116	n/a	0	5	0	1
117	n/a	0	5	0	1
118	n/a	0	5	0	1
119	n/a	0	6	0	1
120	n/a	0	6	0	1
121	n/a	0	5	0	1
122	n/a	0	6	0	1
123	n/a	0	6	0	1
124	n/a	0	7	0	1
125	n/a	0	5.5	0	1
126	n/a	0	6	0	0
127	n/a	0	5	0	0
128	n/a	0	7	0	0
129	n/a	0	6	0	0
130	n/a	0	6	0	0
131	n/a	0	7	0	0
132	n/a	0	6	0	0
133	n/a	0	6	0	0
134	n/a	0	6	0	0
135	n/a	0	7	0	1
136	n/a	0	7	0	1
137	n/a	0	6	0	1
138	n/a	0	6	0	1
139	n/a	0	7	0	1
140	n/a	0	6	0	0
141	n/a	0	4	0	0
142	n/a	0	7	0	1
143	n/a	0	7	0	1
144	n/a	0	7	0	1
145	n/a	0	6	0	1
146	n/a	0	7	0	0
147	n/a	0	7	0	1
148	n/a	0	6	0	1
149	n/a	0	7	0	1
150	n/a	0	7	0	0
151	n/a	0	7	0	0
152	n/a	0	7	0	0
153	n/a	0	4	0	0
154	n/a	0	5	0	1
155	n/a	0	7	0	0
156	n/a	0	6	0	1
157	n/a	0	7	0	0
158	n/a	0	6	0	0
159	n/a	0	6	0	0
160	n/a	0	7	0	0
161	n/a	0	7	0	0
162	n/a	0	7	0	0
163	n/a	0	7	0	1
164	n/a	0	6	0	1
165	n/a	0	7	0	0
166	n/a	0	7	0	0
167	n/a	0	7	0	0
168	n/a	0	7	0	0
169	n/a	0	7	0	1
170	n/a	0	7	0	1
171	n/a	0	7	0	1
172	n/a	0	6	0	1
173	n/a	0	7	0	0
174	n/a	0	7	0	0
175	n/a	0	7	0	0
176	n/a	0	7	0	0
177	n/a	0	7	0	0
178	n/a	0	6	0	0
179	n/a	0	6	0	1
180	n/a	0	5	0	0
181	n/a	0	5	0	1
182	n/a	0	6	0	1
183	n/a	0	6	0	1
184	n/a	0	6	0	1
185	n/a	0	5	0	1
186	n/a	0	6	0	0
187	n/a	0	6	0	0
188	n/a	0	6	0	0
189	n/a	0	5	0	1
190	n/a	0	5	0	1
191	n/a	0	6	0	1
192	n/a	0	6	0	0
193	n/a	0	6	0	1
194	n/a	0	6	0	1
195	n/a	0	6	0	1
196	n/a	0	6	0	0
197	n/a	0	6	0	0
198	n/a	0	6	0	0
199	n/a	0	6	0	0
200	n/a	0	6	0	0
201	n/a	0	7	0	0
202	n/a	0	6	0	1
203	n/a	0	5	0	1
204	n/a	0	5	0	1
205	n/a	0	5	0	1
206	n/a	0	4	0	1
207	n/a	0	5	0	1
208	n/a	0	5	0	1
209	n/a	0	6	0	0
210	n/a	0	6	0	1
211	n/a	0	4	0	1
212	n/a	0	4	0	1
213	n/a	0	5	0	0
214	n/a	0	6	0	0
215	n/a	0	5	0	0
216	n/a	0	6	0	0
217	n/a	0	5	0	0
218	n/a	0	6	0	0
219	n/a	0	7	0	0
220	n/a	0	6	0	1
221	n/a	0	7	0	1
222	n/a	0	6	0	1
223	n/a	0	6	0	1
224	n/a	0	6	0	1
225	n/a	0	6.5	0	1
226	n/a	0	6	0	0
227	n/a	0	5	0	0
228	n/a	0	6	0	0
229	n/a	0	5	0	0
230	n/a	0	6	0	0
231	n/a	0	6	0	0
232	n/a	0	6	0	0
233	n/a	0	6	0	1
234	n/a	0		0	1
235	n/a	0	6	0	1
236	n/a	0	6	0	1
237	n/a	0		0	1
238	n/a	0	6	0	1
239	n/a	0	6	0	0
240	n/a	0	5	0	1
241	n/a	0	6	0	1
242	n/a	0	6	0	1
243	n/a	0	6	0	0
244	n/a	0	6	0	1
245	n/a	0	6	0	1
246	n/a	0	6.5	0	1
247	n/a	0	6	0	1
248	n/a	0	6	0	1
249	n/a	0	6	0	1
250	n/a	0	6	0	1
251	n/a	0	5	0	1
252	n/a	0	4	0	1
253	n/a	0	6	0	1
254	n/a	0	5	0	1
255	n/a	0	6	0	0
256	n/a	0	4	0	1
257	n/a	0	5	0	1
258	n/a	0	5	0	1
259	n/a	0	5	0	0
260	n/a	0	4	0	1
261	n/a	0	5	0	1
262	n/a	0	5	0	1
263	n/a	0	6	0	1
264	n/a	0	5	0	0
265	n/a	0	5	0	0
266	n/a	0	6	0	0
267	n/a	0	6	0	0
268	n/a	0	6	0	0
269	n/a	0	7	0	1
270	n/a	0	6	0	1
271	n/a	0	6	0	0
272	n/a	0	7	0	0
273	n/a	0	5	0	1
274	n/a	0	6	0	1
275	n/a	0	4	0	1
276	n/a	0	4	0	1
277	n/a	0	5	0	1
278	n/a	0	5	0	1
279	n/a	0	7	0	1
280	n/a	0	6	0	1
281	n/a	0	5	0	1
282	n/a	0	5	0	0
283	n/a	0	6	0	0
284	n/a	0	6	0	0
285	n/a	0	6	0	0
286	n/a	0	6	0	0
287	n/a	0	6	0	0
288	n/a	0	5	0	0
289	n/a	0	5	0	0
290	n/a	0	5	0	0
291	n/a	0	6	0	0
292	n/a	0	5	0	0
293	n/a	0	4	0	0
294	n/a	0	6	0	0
295	n/a	0	6	0	0
296	n/a	0	6	0	0
297	n/a	0	5	0	0
298	n/a	0	6	0	0
299	n/a	0	7	0	0
300	n/a	0	6	0	0
301	n/a	0	6	0	0
302	n/a	0	7	0	0
303	n/a	0	6	0	0
304	n/a	0	7	0	0
305	n/a	0	6	0	0
306	n/a	0	6	0	0
307	n/a	0	5	0	1
308	n/a	0	5	0	1
309	n/a	0	5	0	1
310	n/a	0	5	0	1
311	n/a	0	6	0	0
312	n/a	0	4	0	0
313	n/a	0	5	0	0
314	n/a	0	6	0	0
315	n/a	0	6	0	0
316	n/a	0	6	0	0
317	n/a	0	7	0	0
318	n/a	0	7	0	0
319	n/a	0	6	0	0
320	n/a	0	6	0	0
321	n/a	0	6	0	1
322	n/a	0	6	0	0
323	n/a	0	7	0	0
324	n/a	0	7	0	0
325	n/a	0	4.5	0	1
326	n/a	0	5	0	1
327	n/a	0	6	0	1
328	n/a	0	6	0	1
329	n/a	0	6	0	1
330	n/a	0	5	0	1
331	n/a	0	5.5	0	1
332	n/a	0	5	0	1
333	n/a	0	5	0	1
334	n/a	0	6	0	1
335	n/a	0	6	0	1
336	n/a	0	4	0	1
337	n/a	0	5	0	0
338	n/a	0	4	0	1
339	n/a	0	5	0	1
340	n/a	0	5	0	1
341	n/a	0	6	0	1
342	n/a	0	6	0	1
343	n/a	0	4	0	1
344	n/a	0	4	0	0
345	n/a	0	6	0	0
346	n/a	0	6	0	0
347	n/a	0	6	0	0
348	n/a	0	7	0	1
349	n/a	0	6	0	1
350	n/a	0	6	0	1
351	n/a	0	6	0	1
352	n/a	0	5	0	1
353	n/a	0	5	0	0
354	n/a	0	5	0	1
355	n/a	0	6	0	1
356	n/a	0	5	0	0
357	n/a	0	5	0	1
358	n/a	0	5	0	1
359	n/a	0	5	0	1
360	n/a	0	6	0	1
361	n/a	0	5	0	1
362	n/a	0	6	0	1
363	n/a	0	6	0	0
364	n/a	0	7	0	0
365	n/a	0	7	0	0
366	n/a	0	6	0	1
367	n/a	0	6	0	0
368	n/a	0	6	0	0
369	n/a	0	6	0	0
370	n/a	0	6	0	0
371	n/a	0	6	0	0
372	n/a	0	5	0	0
373	n/a	0	6	0	0
374	n/a	0	7	0	0
375	n/a	0	5	0	0
376	n/a	0	5	0	0
377	n/a	0	6	0	0
378	n/a	0	6	0	0
379	n/a	0	6	0	0
380	n/a	0	7	0	0
381	n/a	0	7	0	0
382	n/a	0	6	0	0
383	n/a	0	6	0	0
384	n/a	0	6	0	0
385	n/a	0	6	0	0
386	n/a	0	6	0	0
387	n/a	0	6	0	0
388	n/a	0	6	0	0
389	n/a	0	6	0	0
390	n/a	0	6	0	0
391	n/a	0	6	0	0
392	n/a	0	6	0	0
393	n/a	0	6	0	0
394	n/a	0	5	0	0
395	n/a	0	5	0	0
396	n/a	0	6	0	0
397	n/a	0	7	0	1
398	n/a	0	7	0	1
399	n/a	0	5	0	0
400	n/a	0	7	0	0
401	n/a	0	6	0	0
402	n/a	0	7	0	1
403	n/a	0	5	0	1
404	n/a	0	7	0	1
405	n/a	0	6	0	1
406	n/a	0	6	0	1
407	n/a	0	7	0	1
408	n/a	0	7	0	1
409	n/a	0	7	0	1
410	n/a	0	7	0	0
411	n/a	0	7	0	1
412	n/a	0	5	0	0
413	n/a	0	6	0	0
414	n/a	0	5	0	0
415	n/a	0	4	0	0
416	n/a	0	6	0	1
417	n/a	0	6	0	1
418	n/a	0	6	0	1
419	n/a	0	7	0	1
420	n/a	0	5	0	1
421	n/a	0	6	0	1
422	n/a	0	6	0	1
423	n/a	0	7	0	1
424	n/a	0	7	0	1
425	n/a	0	6	0	1
426	n/a	0	5	0	1
427	n/a	0	5	0	1
428	n/a	0	6	0	1
429	n/a	0	6	0	1
430	n/a	0	5	0	1
431	n/a	0	6	0	1
432	n/a	0	5	0	1
433	n/a	0	5	0	1
434	n/a	0	6	0	1
435	n/a	0	6	0	1
436	n/a	0	6	0	1
437	n/a	0	6	0	1
438	n/a	0	6	0	0
439	n/a	0	6	0	1
440	n/a	0	6	0	1
441	n/a	0	6	0	1
442	n/a	0	6	0	1
443	n/a	0	7	0	1
444	n/a	0	6	0	1
445	n/a	0	6	0	1
446	n/a	0	6	0	1
447	n/a	0	7	0	1
448	n/a	0	7	0	0
449	n/a	0	5	0	0
450	n/a	0	6	0	0
451	n/a	0	5	0	0
452	n/a	0	7	0	0
453	n/a	0	6	0	0
454	n/a	0		0	1
455	n/a	0		0	0
456	n/a	0		0	0
457	n/a	0	7	0	1
458	n/a	0	6	0	1
459	n/a	0	6	0	1
460	n/a	0	6	0	1
461	n/a	0	6	0	1
462	n/a	0	4	0	1
463	n/a	0	5	0	0
464	n/a	0	6	0	0
465	n/a	0	6	0	0
466	n/a	0	6	0	0
467	Excellent	1	6	0	1
468	?Rushes	1	6	0	1
469	None	1	6	0	1
470	None	1	6	0	1
471	Good!!!	1	7	0	0
472	nil	1	7	0	1
473	Excellent	1	6	0	0
474	Awesome.	1	7	0	1
475	able	1	6	0	0
476	Great	1	7	0	0
477	Accurate	1	6	1	1
478	Excellent!	1	6	0	1
479	none	1	7	0	1
480	Superlative	1	7	0	0
481	Exceptional	1	7	0	0
482	Solid	1	6	0	1
483	No concerns.	2	6	0	0
484	Excellent H/P/P/A	2	5	0	1
485	Progressing well	2	6	0	1
486	Excellent work	2	6	0	1
487	See previous	2	5	0	0
488	No concerns	2	6	0	1
489	no concerns	2	6	0	0
490	no concerns	2	6	0	0
491	no concerns	2	6	0	0
492	as above	2	6	0	0
493	Neonatal jaundice	2	6	1	1
494	Document assessment	2	6	0	0
495	see prev.	2	6	0	0
496	See previous	2	6	0	0
497	No weaknesses	2	6	0	1
498	See previous.	2	6	0	0
499	see above..	2	7	0	0
500	see prev.	2	7	0	0
501	No concerns	2	5	0	1
502	No concerns.	2	5	0	0
503	No concerns	2	6	0	1
504	Excellent resident	2	7	0	1
505	as above	2	6	0	0
506	Good resident	2	7	0	0
507	as above	2	7	0	0
508	Strong resident!	2	7	0	1
509	Excellent resident	2	7	0	1
510	Simply outstanding !!!	2	7	0	0
511	No concerns.	2	7	0	1
512	Excellent job	2	7	0	1
513	overall efficient	2	6	0	1
514	overall good.	2	6	0	0
515	No concern	2	7	0	1
516	Excellent resident!	2	7	0	1
517	As above	2	6	0	0
518	See above	2	7	0	0
519	Works independently	2	5.5	0	1
520	Works independently	2	5	1	1
521	Works efficiently	2	6	1	1
522	Works independently	2	6	0	1
523	Strong RI	2	6	0	1
524	Efficient historian.	2	6	0	1
525	Great documentation	2	7	0	1
526	See above	2	7	0	0
527	Efficient, thorough.	2	7	0	0
528	Great charting	2	6	1	1
529	Excellent intubation	2	6	1	0
530	Hard worker	2	4	1	1
531	Progressing well	2	6	0	1
532	Good resident	2	6	1	1
533	Excellent resident	2	7	0	1
534	Good instructions	2	6	0	1
535	Excellent resident	2	7	0	1
536	Hard worker	2	6	0	1
537	Working well	2	6	0	0
538	Excellent resident	2	6	0	0
539	No concerns	2	6	0	0
540	meets criteria	2	7	0	0
541	Good shift	2	7	0	1
542	As above.	2	7	0	0
543	Great shift	2	7	0	0
544	As above.	2	7	0	0
545	Become PGY3!	2	7	0	1
546	No concerns	2	5	0	1
547	As above	2	6	0	0
548	Excellent resident	2	6	0	0
549	Good resident.	2	6	0	0
550	see above	2	6	0	0
551	see above	2	5	0	0
552	progressing well	2	6	0	0
553	good assessments	2	6	0	0
554	meets requirements	2	7	0	0
555	Good assessments.	2	5	0	0
556	Functions well	2	6	0	0
557	excellent assessments	2	6	0	0
558	good shift	2	6	0	0
559	strong resident	2	6	0	0
560	Well done.	2	6	0	0
561	competent resident	2	6	0	0
562	Technologically sound	2	7	0	1
563	Doing well.	2	7	0	0
564	Excellent work	2	6	0	1
565	Awesome work!	2	7	0	1
566	Excellent work!	2	7	0	1
567	Excellent job.	2	6	0	0
568	Well done.	2	6	0	0
569	Good resident	2	6	0	1
570	Overall good.	2	6	0	0
571	Great suturing!	2	6	0	1
572	Appropriate investigations	2	5	0	1
573	No concerns.	2	6	0	0
574	doing well	2	6	0	0
575	doing well	2	7	0	0
576	doing ok	2	5	0	0
577	as above	2	7	0	0
578	As above	2	7	0	0
579	as above	2	7	0	0
580	as above	2	7	0	0
581	as above	2	7	0	0
582	Good charting	2	7	1	1
583	No concerns	2	6	0	1
584	Competent/efficient	2	7	0	1
585	Well done	2	6	0	1
586	Solid shift	2	7	0	0
587	As above	2	4	0	0
588	Strong resident.	2	6	0	0
589	as above.	2	5	0	0
590	as previous.	2	7	0	0
591	As before.	2	7	0	0
592	solid shift.	2	7	0	0
593	Great job.	2	7	0	0
594	No concerns.	2	6	0	0
595	Good shift	2	5	0	0
596	excellent shift	2	6	0	0
597	done previously	2	7	0	0
598	Good assessments	2	6	1	1
599	Excellent assessment	2	7	1	1
600	Independent, knowledgeable	2	6	0	1
601	Functions independently	2	6	0	1
602	Good assessments	2	6	0	1
603	Excellent assessments	2	7	0	1
604	Great assessments.	2	5	1	0
605	Good job	2	6	0	1
606	See previous	2	6	0	0
607	As above	2	6	0	0
608	See previous	2	6	0	0
609	Pleasant, motivated	2	6	0	1
610	Excellent shift	2	7	0	1
611	Excellent shift	2	7	0	1
612	Excellent shift	2	7	0	1
613	Excellent shift	2	7	0	1
614	Excellent shift	2	7	0	1
615	Excellent shift	2	7	0	1
616	Excellent shift	2	6	0	0
617	Consistently professional	2	5.8	0	1
618	doing well	2	6	0	0
619	See consultation comments	3	5	0	0
620	Excellent work today	3	4	0	1
622	Excellent efficient resident.	3	6	1	0
623	My first one	3	7	0	0
624	See previous note	3	6	0	0
625	done as above	3	7	0	0
626	Good shift. Busy.	3	6	0	1
627	As previously noted	3	6	0	0
628	Functions well independently.	3	5	0	0
629	Very enthusiastic. Hardworking	3	6	0	1
630	Document pertinent negatives	3		1	0
631	Good differential diagnosis	3	6	0	0
632	Good presentation skills	3	5	1	0
633	see prev. explanation..	3	6	0	0
634	appropriate for level	3	5	0	0
635	Good patient advocacy	3	7	1	0
636	Excellent, excellent resident!	3	7	0	1
637	See previous comments	3	7	0	0
638	Doing very well.	3	6	0	0
639	Excellent management plans.	3	6	0	0
640	Great! Superior resident	3	7	0	1
641	Functions independently, efficient	3	6	0	1
642	good resident independent	3	6	0	0
643	Runs department effectively	3	7	0	0
644	Independent and confident.	3	7	1	0
645	Efficient consent obtained	3	7	1	1
646	No concerns. Excellent	3	6	0	1
647	Appropriate for senior	3	7	0	1
648	Proactive and enthusiastic	3	6	0	1
649	Review toxic alcohols	3	6	1	1
650	pleasure, developing nicely.	3	6	0	0
651	Not grossly unsatisfactory.	3	7	0	0
652	Hardworking, no concerns	3	6	0	1
653	Very good resident	3	6	0	1
654	Student progress well	3	5	0	1
655	Hard working resident	3	7	0	1
656	hard working resident,	3	6	1	0
657	Continues? comprehensive assessment	3	6	1	1
658	Consistently good performance	3	6	0	1
659	Structure differential/plan	3	4	1	1
660	Short ?? ??	3	6	0	1
661	Organized/appropriate plans	3	5	0	1
662	Follow-up/reassess ??	3	4	0	1
663	Good overall performance	3	5	0	0
664	Consistent comprehensive performance	3	6	1	0
665	Well done. Excellent.	3	7	0	1
666	Go to PGY3	3	7	0	1
667	Time for PGY3	3	7	0	1
668	Very strong resident	3	6	1	0
669	Very good resident.	3	6	0	0
670	Very good resident.	3	6	0	0
671	Very good resident	3	6	0	0
672	Overall very good.	3	6	0	0
673	Good job today	3	5	0	1
674	Great job today!	3	6	0	0
675	Independent with procedure	3	7	0	0
676	strong clinical skills	3	6	0	0
677	Excellent patient care.	3	7	1	0
678	Great work today!	3	6	0	1
679	Efficient, independent, multitasking.	3	6	0	0
680	Overall excellent resident	3	6	0	1
681	Overall great resident	3	6.5	0	1
682	Overall very good	3	6	0	0
683	continuing to improve	3	5	0	0
684	Excellent. Dependable. Trustworthy.	3	7	0	0
685	Very strong resident.	3	7	0	0
686	A good day	3	7	0	1
687	Another great shift	3	7	0	1
688	A good shift.	3	6	0	0
689	not completed today	3	5	0	0
690	No real concerns	3	6	0	0
691	Managed volume well	3	7	0	1
692	Very good assessments	3	6	1	1
693	Very good assessments	3	6	1	1
694	Better organized today	3	6	0	1
695	Read around cases	3	5	0	1
696	functions very well.	3	6	0	0
697	Good ER shift	3	7	0	1
698	Excellent ?? shift	3	7	0	1
699	Excellent mgmt/presentation	3	7	0	1
700	Appropriate for level	3	4	0	1
701	Excellent overall shift	3	7	0	1
702	Good shift today	3	7	0	1
703	Very good shift	3	6	0	1
704	Appropriate for PGY2	3	5	0	0
705	Excellent overnight shift...	3	6	0	0
706	Excellent independent resident	3	7	0	1
708	Carried dept - mostly	3	7	1	0
709	doing very well	3	6	0	0
710	continues to progress	3	6	0	0
711	MOTIVATED, WORKS HARD	3	6	1	0
712	did very well	3	6	0	0
713	worked independently well	3	7	0	0
		Mode	6	0	0
				Average=0.08	Count paper=338
					Count electronic=373
